# Outcomes of Lung Transplantation in Patients With Right Ventricular Dysfunction: A Single-Center Retrospective Analysis Comparing ECMO Configurations in a Bridge-to-Transplant Setting

**DOI:** 10.3389/ti.2024.12657

**Published:** 2024-05-23

**Authors:** Su Yeon Lee, Jee Hwan Ahn, Ho Cheol Kim, Tae Sun Shim, Pil-Je Kang, Geun Dong Lee, Se Hoon Choi, Sung-Ho Jung, Seung-Il Park, Sang-Bum Hong

**Affiliations:** Asan Medical Center, College of Medicine, University of Ulsan, Seoul, Republic of Korea

**Keywords:** lung transplant, ECMO, right ventricular dysfunction, bridge to transplant, survival outcomes

## Abstract

This study aimed to assess the lung transplantation (LT) outcomes of patients with right ventricular dysfunction (RVD), focusing on the impact of various extracorporeal membrane oxygenation (ECMO) configurations. We included adult patients who underwent LT with ECMO as a bridge-to-transplant from 2011 to 2021 at a single center. Among patients with RVD (*n* = 67), veno-venous (V-V) ECMO was initially applied in 79% (53/67) and maintained until LT in 52% (35/67). Due to the worsening of RVD, the configuration was changed from V-V ECMO to veno-arterial (V-A) ECMO or a right ventricular assist device with an oxygenator (Oxy-RVAD) in 34% (18/67). They showed that lactic acid levels (2–6.1 mmol/L) and vasoactive inotropic score (6.6–22.6) increased. V-A ECMO or Oxy-RVAD was initiated and maintained until LT in 21% (14/67) of cases. There was no significant difference in the survival rates among the three configuration groups (V-V ECMO vs. configuration changed vs. V-A ECMO/Oxy-RVAD). Our findings suggest that the choice of ECMO configuration for LT candidates with RVD should be determined by the patient’s current hemodynamic status. Vital sign stability supports the use of V-V ECMO, while increasing lactic acid levels and vasopressor needs may require a switch to V-A ECMO or Oxy-RVAD.

## Graphical Abstract

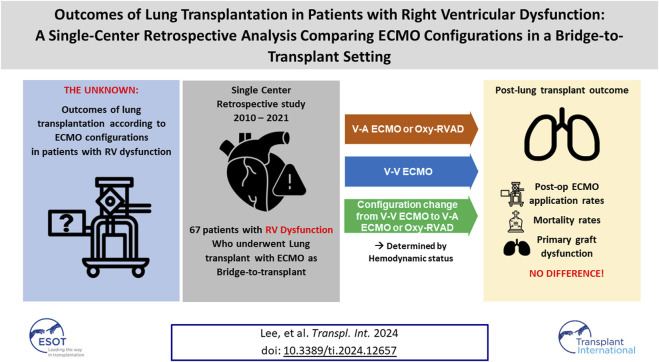

## Introduction

Lung transplantation has become the standard treatment for end-stage lung disease [1]. Many patients awaiting lung transplantation develop right ventricular (RV) dysfunction due to pulmonary hypertension, a consequence of their disease’s progression. Secondary pulmonary hypertension is present in up to 66% of patients with end-stage pulmonary disease, particularly reaching up to 84% in patients with idiopathic pulmonary fibrosis [2, 3]. The 2021 guidelines from the International Society for Heart and Lung Transplant recommend listing patients for lung transplantation when they show signs of pulmonary hypertension or RV failure, as these conditions are associated with poorer outcomes [4]. RV systolic dysfunction is also recognized as a risk factor for mortality in patients using extracorporeal membrane oxygenation (ECMO) as a bridge to transplantation (BTT) [5]. Therefore, recognizing and managing patients with RV dysfunction effectively is important, placing a special focus on maintaining appropriate volume and hemodynamic support [6].

Mechanical circulatory support strategies while awaiting lung transplantation may differ between patients with end-stage lung disease with secondary pulmonary hypertension and primary pulmonary hypertension based on the severity of their pulmonary failure and cardiac dysfunction [6, 7]. In 2022, the American Association for Thoracic Surgery (AATS) published an expert consensus document on the use of mechanical circulatory support in lung transplantation [8]. According to this consensus, veno-arterial (V-A) ECMO can be a valuable option as a BTT for end-stage pulmonary hypertension with RV failure. However, not all the patients with RV dysfunction were on V-A ECMO at the time of the first ECMO initiation. Research comparing the outcomes of different ECMO configurations in patients with RV dysfunction is limited, and there is no established study on when to initiate V-A ECMO. Also, it is well reported that V-A ECMO can present more complications than V-V ECMO, including a heightened risk of bleeding issues such as surgical site, abdominal, and retroperitoneal hemorrhages, as well as potentially devastating complications, such as systemic thromboembolism-related neurological complications, and limb ischemia [9, 10]. Therefore, the present study aimed to (1) evaluate the lung transplantation outcomes of BTT patients with RV dysfunction, focusing on their specific ECMO configurations, and (2) identify characteristics including timing, lactic acid levels, and vasoactive inotropic doses when initiating V-A ECMO or RV assist devices with an oxygenator (Oxy-RVAD) in patients with RV dysfunction.

## Materials and Methods

### Study Patients and Designs

**FIGURE 1:**
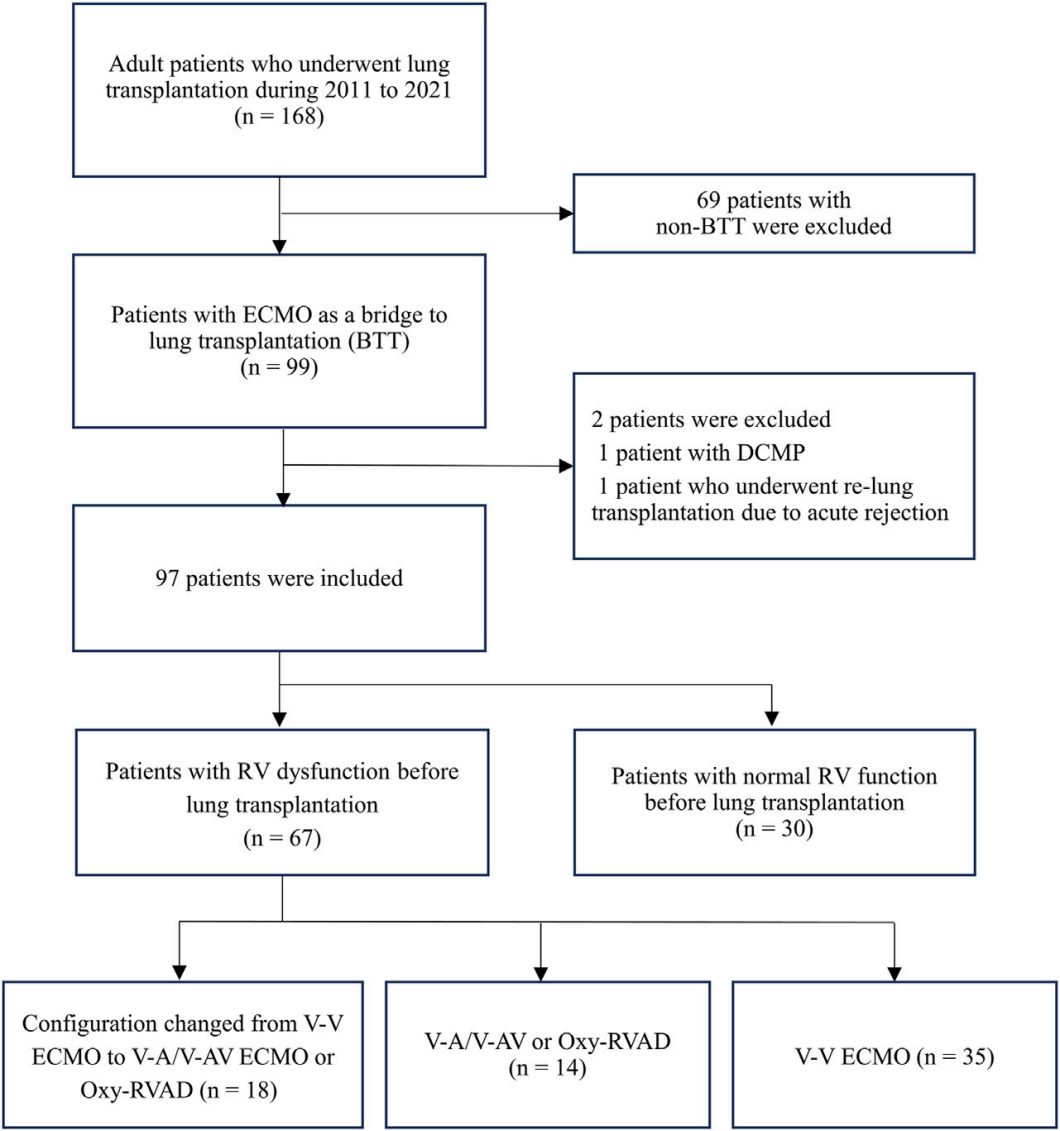
Diagram of patient inclusion flow Abbreviations: ECMO, extracorporeal membrane oxygenation; DCMP, dilated cardiomyopathy; RV, right ventricular; V-V, Veno-venous; V-A, Veno-arterial; V-AV, Veno-arteriovenous; Oxy-RVAD, right ventricular assist device with and oxygenator.

This retrospective cohort study was conducted at a single-center tertiary hospital in Korea. Among the adult patients (age >18 years) who underwent lung transplantation between 2011 and 2021, those with ECMO as BTT were enrolled in the present study (Figure 1). We excluded patients with left ventricular (LV) dysfunction or dilated cardiomyopathy (DCMP) and those who underwent retransplantation due to acute rejection. In all patients, we reviewed the results of preoperative transthoracic echocardiography (TTE) conducted nearest to the date of lung transplantation, and data on RV dysfunction by the official note of cardiologists were collected. RV systolic dysfunction was assessed according to the guidelines of the American Society of Echocardiography [11, 12]. Visual assessment of free wall contractility was performed in all patients, and parameters such as fractional area change (FAC) < 35%, tricuspid annular plane systolic excursion (TAPSE) < 16 mm, tissue Doppler-derived tricuspid lateral annular systolic velocity (S’) < 10 cm/s, right ventricular index of myocardial performance (RIMP) > 0.4 by pulsed Doppler, and >0.55 by tissue Doppler were used to define RV dysfunction.

### ECMO Strategies in Lung Transplant Candidates

Due to the shortage of lung donors in South Korea, many patients undergo lung transplantation in status 0, which refers to patients on mechanical ventilation or ECMO [13, 14]. When patients on the waiting list develop respiratory failure, they are intubated, started on mechanical ventilation, and uplisted to status 0. If oxygenation and ventilation cannot be maintained by mechanical ventilation alone, and if patients are hemodynamically stable, V-V ECMO is then used [9]. In our center, the right femoral-right internal jugular vein was mainly selected for V-V ECMO configuration. Intensive care physicians routinely performed focused cardiac ultrasound [15], and monitored cardiac enzymes and BNP levels regularly from the time patients were admitted to the intensive care unit (ICU). If patients were hemodynamically unstable with lactic acid levels greater than 4 mmol/L or norepinephrine levels greater than 0.2 mcg/kg/min (or equivalent doses of epinephrine or dobutamine), V-A/V-AV ECMO or Oxy-RVAD was considered after a multidisciplinary team review. While the femoral vein and femoral arterial cannula were usually used in V-A ECMO, an additional femoral arterial cannula was inserted to change the configuration from V-V ECMO to V-AV ECMO. For Oxy-RVAD, a left anterior thoracotomy was performed to place the reinfusion cannula in the pulmonary artery. A bolus of unfractionated heparin (50–70 units/kg) was infused at the start of ECMO support, and heparin was continuously infused to achieve an activated partial thromboplastin time of 40–60 s during V-V ECMO support and 50–75 s during V-A ECMO support. Once patients achieve hemodynamic stabilization, preoperative rehabilitation begins with both mechanical ventilation with early tracheostomy and ECMO support [16]. A few patients underwent lung transplantation as awakening ECMO. If patients with V-V ECMO experienced hemodynamic instability during rehabilitation, the configuration was changed to Oxy-RVAD or V-A/V-AV ECMO. When peripheral V-A ECMO or V-AV ECMO impeded rehabilitation due to cannula position, conversion to central V-A ECMO or Oxy-RVAD was considered [17, 18]. Postoperative ECMO has been used in patients with unstable vital signs or hypoxemia at the end of surgery [6].

### Data Collection

We collected the following patient data from the electronic medical records: age, sex, ABO types, height, weight, body mass index (BMI), underlying disease, date of lung transplantation, dates of hospital and ICU admission and discharge, date of death, status of preoperative rehabilitation; TTE results before lung transplantation: left ventricular ejection fraction (LVEF), the ratio of early diastolic mitral inflow velocity to early diastolic mitral annular tissue velocity (E/E’), maximal tricuspid regurgitation velocity (TR Vmax), systolic pressure gradient between the right ventricle and right atrium (RV-RA pressure gradient); Troponin-I, brain natriuretic peptide (BNP), simplified acute physiology score (SAPS) II at lung transplantation day, preoperative and postoperative vasopressor types and doses, dates of mechanical ventilation, date of tracheostomy, ECMO types and configurations, date of ECMO insertion, date of ECMO configuration change, date of ECMO discontinuation. Primary graft dysfunction (PGD) at the 72-h time point was recorded [19]. We reviewed complications during ECMO such as bleeding, ECMO pump clots, ECMO cannula site complications, and continuous renal replacement therapy. We defined “bleeding” as any bleeding site such as gastrointestinal, retroperitoneal, intracranial, tracheostomy site, cannula site, hemoptysis, hemothorax, etc., including major to minor bleeding [20]. We defined “ECMO cannula site complications” as ECMO catheter thrombosis, and wound complications such as wound infection. To quantify the severity of hemodynamic instability, this study measured the Vasoactive Inotropic Score and lactic acid. The vasoactive inotropic score (VIS), which is a weighted sum of all administered vasoactive inotropic agents [21], was calculated just before ECMO insertion, and ECMO change. The lactic acid levels were checked at the same time.

### Statistical Analysis

Variables are presented either as means with standard deviations or medians with interquartile ranges, based on their distribution. The included patients were divided into the RV dysfunction group and the normal RV function group. Patients with RV dysfunction were further divided into three groups: 1) V-V ECMO maintenance, 2) configuration change from V-V ECMO to V-A/V-AV or Oxy-RVAD, and 3) V-A/V-AV ECMO or Oxy-RVAD maintenance group. Baseline characteristics and outcomes of patients were compared according to their RV function and ECMO configuration. In addition, VIS and lactic acid levels were compared between initial ECMO insertion and ECMO configuration change. For the comparison of continuous variables, we used the student’s t-test. The chi-squared or Fisher’s exact tests were chosen for categorical variables. Survival analyses were performed using the Kaplan–Meier method and the log-rank test. All *p*-values were two-tailed, with the threshold for statistical significance set at a *p*-value of <0.05. All statistical analyses and graphs were conducted using R 4.2.1 (R Core Team, Vienna, Austria).

### Ethics Approval and Consent to Participate

The study protocol was reviewed and approved by the Institutional Review Board of our center (IRB number: 2019-0981, approval date: 2019-08-02), which waived the requirement for obtaining patient informed consent due to the observational nature of this study.

## Results

Ninety-seven patients who underwent lung transplantation with BTT between 2011 and 2021 were analyzed. Their median days from TTE to lung transplantation was 8 [0.5–15.5] days and most patients had a TTE test within a month from lung transplantation. Of the 97 patients with BTT, 69% (*n* = 67) had documented RV dysfunction and 31% (*n* = 30) had normal RV function prior to lung transplantation (Figure 1). Among patients with RV dysfunction (*n* = 67), 53 patients (79%) started V-V ECMO and 14 patients (21%) started V-A/V-AV ECMO or Oxy-RVAD. Thirty-five patients (52%) maintained V-V ECMO, and 18 patients (27%) switched from V-V ECMO to V-A/V-AV ECMO or Oxy-RVAD while awaiting lung transplantation.

Among the BTT patients (*n* = 97), their median age was 58 years and blood type A was the most common type (35.1%) (Table 1). Interstitial lung disease (ILD) patients were the most common (77.3%), and bleeding (39.2%) was the most common complication observed in patients with BTT. When the data between the groups with RV dysfunction and normal RV function were compared, preoperative TTE showed higher TR Vmax (3.4 ± 0.7 vs. 2.8 ± 0.5 m/s, *p* < 0.001) and higher RV-RA pressure gradient (49.0 ± 16.7 vs. 33.5 ± 11.6 mmHg, *p* < 0.001) in the RV dysfunction group. The lactic acid level (3.5 ± 3.3 vs. 2.1 ± 2.5 mmol/L, *p* = 0.039) and VIS (12.5 ± 16.7 vs. 4.9 ± 10.5, *p* < 0.008) at ECMO initiation were also higher in the RV dysfunction group than in the normal RV function group. When the date of lung transplantation, SAPS II was higher (35.7 ± 12.5 vs. 31.1 ± 6.9, *p* = 0.022) and BNP was also higher (537.3 ± 699.6 vs. 122.7 ± 147.5 pg/mL) in patients with RV dysfunction.

**TABLE 1:** Characteristics of the patients with and without RV dysfunction before lung transplantation among bridge-to-transplant patients.

	Normal RV function (*n* = 30)	RV dysfunction (*n* = 67)	Total (*n* = 97)	*p*-value
Age, median [IQR]	56.5 [44.0; 63.0]	58.0 [49.0; 63.0]	58.0 [47.0; 63.0]	0.625
Male sex, n (%)	15 (50.0)	25 (37.3)	40 (41.2)	0.342
ABO types, n (%)				0.792
A	12 (40.0)	22 (32.8)	34 (35.1)	
B	7 (23.3)	20 (29.9)	27 (27.8)	
O	4 (13.3)	12 (17.9)	16 (16.5)	
AB	7 (23.3)	13 (19.4)	20 (20.6)	
BMI, kg/m^2^, mean ± SD	21.5 ± 3.1	23.5 ± 4.5	22.9 ± 4.2	0.012
Diagnosis, n (%)				
ILD	24 (80.0)	51 (76.1)	75 (77.3)	0.598
BO	1 (3.3)	2 (3.0)	3 (3.1)	
ARDS	5 (16.7)	10 (14.9)	15 (15.5)	
PAH	0 (0.0)	4 (6.0)	4 (4.1)	
Preop rehabilitation, n (%)	22 (73.3)	52 (77.6)	74 (76.3)	0.842
Standing	10 (33.3)	23 (34.3)	33 (34.0)	0.505
Dangling	2 (6.7)	1 (1.5)	3 (3.1)	0.505
Bed exercise	10 (33.3)	28 (41.8)	38 (39.2)	0.505
Transthoracic echocardiography				
LVEF, %, mean ± SD	63.9 ± 6.4	60.6 ± 7.8	61.6 ± 7.5	0.046
E/E′, mean ± SD	8.9 ± 1.4	8.8 ± 3.6	8.9 ± 3.1	0.916
TR Vmax, m/s, mean ± SD	2.8 ± 0.5	3.4 ± 0.7	3.2 ± 0.7	<0.001
PGsys(RV-RA), mmHg, mean ± SD	33.5 ± 11.6	49.0 ± 16.7	44.4 ± 16.8	<0.001
Lactic acid at ECMO insertion, mmol/L, mean ± SD	2.1 ± 2.5	3.5 ± 3.3	3.1 ± 3.1	0.039
VIS at ECMO insertion, mean ± SD	4.9 ± 10.5	12.5 ± 16.7	10.2 ± 15.4	0.008
Preop ECMO duration days, median [IQR]	12.5 [5.0; 22.0]	14.0 [6.5; 27.0]	13.0 [6.0; 24.0]	0.631
Complications during ECMO, (%)	12 (40.0)	34 (50.7)	46 (47.4)	0.447
Bleeding	12 (40.0)	26 (38.8)	38 (39.2)	1.000
ECMO pump clot	1 (3.3)	14 (20.9)	15 (15.5)	0.056
ECMO site complications	2 (6.7)	11 (16.4)	13 (13.4)	0.327
CRRT	0 (0.0)	9 (13.4)	9 (9.3)	0.084
SAPS II at operation day, mean ± SD	31.1 ± 6.9	35.7 ± 12.5	34.2 ± 11.2	0.022
BNP at operation day, pg/mL, mean ± SD	122.7 ± 147.5	537.3 ± 699.6	409.1 ± 616.5	<0.001
Troponin-I at operation day, ng/mL, mean ± SD	0.2 ± 0.6	0.4 ± 0.9	0.3 ± 0.8	0.209

Abbreviations: RV, right ventricular; BMI, body mass index; ILD, interstitial lung disease; BO, bronchiolitis obliterans; ARDS, acute respiratory distress syndrome; PAH, pulmonary arterial hypertension; LVEF, left ventricular ejection fraction; E/E’, the ratio of early diastolic mitral inflow velocity to early diastolic mitral annular tissue velocity; TR, vmax, maximal tricuspid regurgitation velocity; PGsys(RV-RA), systolic pressure gradient between right ventricle and right atrium; ECMO, extracorporeal membrane oxygenation; VIS, vasoactive inotropic score; SAPS, the simplified acute physiology score; CRRT, continuous renal replacement therapy; SD, standard deviation; IQR, interquartile range.

Among the BTT patients, 12 patients (12.4%) received ECMO after lung transplantation and patients stayed in the ICU for a median duration of 22 days after lung transplantation (Table 2). The 1-year, 3-year, and 5-year crude mortality rates were 21.6%, 33.3%, and 58.0%, respectively. When we compared the postoperative ECMO application rates and the grade and percentage of PGD, we did not observe significant differences according to the presence of RV dysfunction.

**TABLE 2:** Post-lung transplantation outcomes of the patients stratified based on RV dysfunction among bridge-to-transplant patients.

	Normal RV function (*n* = 30)	RV dysfunction (*n* = 67)	Total (*n* = 99)	*p*-value
Postop ECMO application, n (%)	5 (16.7)	7 (10.4)	12 (12.4)	0.599
Postop ECMO duration days, median [IQR]	4 [4.0; 5.0]	6.0 [ 3.5; 8.5]	4.5 [3.5; 6.0]	0.324
Postop ICU days, median [IQR]	23.0 [15.0; 36.0]	21.0 [12.0; 35.5]	22.0 [14.0; 36.0]	0.514
Postop hospital days, median [IQR]	88.0 [47.0; 137.0]	79.0 [47.0; 157.5]	82.0 [46.5; 150.0]	0.971
Discharge from hospital, n (%)	27 (90.0)	52 (77.6)	79 (81.4)	0.243
30-day vasoactive drug-free day, median [IQR]	26.0 [24.0; 28.0]	27.0 [22.0; 28.0]	27.0 [23.0; 28.0]	0.997
30-day ventilator-free day, median [IQR]	18.0 [0.0; 24.0]	16.0 [0.0; 24.0]	17.0 [0.0; 24.0]	0.782
30-day mortality, n (%)	2 (6.7)	4 (6.0)	6 (6.2)	1.000
90-day mortality, n (%)	2 (6.7)	8 (11.9)	10 (10.3)	0.668
1-year mortality, n (%)	4/30 (13.3)	17/67 (25.4)	21/97 (21.6)	0.287
3-year mortality, n (%)	4/18 (22.2)	20/54 (37.0)	24/72 (33.3)	0.386
5-year mortality, n (%)	5/10 (50.0)	24/40 (60.0)	29/50 (58.0)	0.830
PGD at T72, n (%)				0.274
Grade 0	15 (50.0)	39 (58.2)	54 (55.7)	
Grade 1	4 (13.3)	9 (13.4)	13 (13.4)	
Grade 2	4 (13.3)	13 (19.4)	17 (17.5)	
Grade 3	7 (23.3)	6 (9.0)	13 (13.4)	

Abbreviations: RV, right ventricular; ECMO, extracorporeal membrane oxygenation; PGD, at T72, primary graft dysfunction at 72-h time point; IQR, interquartile range.

We compared the baseline characteristics of the patients grouped according to their ECMO configurations (Table 3). All idiopathic pulmonary arterial hypertension (PAH) patients were in the V-A/V-AV ECMO or Oxy-RVAD groups. The lactic acid level (8.2 ± 3.5 mmol/L) and VIS (32.7 ± 20.4) were significantly higher in the V-A/V-AV ECMO or Oxy-RVAD group at the initial ECMO insertion (*p* < 0.001 for each). The complication rate was the lowest (37.1%) in the V-V ECMO group and the highest (78.6%) in the V-A/V-AV or Oxy-RVAD group (*p* = 0.029). Preoperative ECMO duration was similar between the V-A/V-AV ECMO or Oxy-RVAD and configuration change groups (16 [13–30] vs. 17 [9–28]). The median duration from starting ECMO to changing the configuration was 7 days [4.3–62.0 days] in the configuration change group. BNP on the operative day (1,114.5 ± 1,133.0 pg/mL) was significantly higher in the V-A/V-AV ECMO or Oxy-RVAD group (*p* = 0.001).

**TABLE 3:** Characteristics of patients according to the ECMO configurations before lung transplantation among bridge-to-transplant patients.

	Initial V-V ECMO (*n* = 53)		V-A ECMO or Oxy-RVAD maintained^b^ (*n* = 14)	
	V-V ECMO maintained (*n* = 35)	Configuration change to V-A/V-AV ECMO or Oxy-RVAD^a^ (*n* = 18)		*p*-value
Age, median [IQR]	60.0 [52.0; 63.0]	59.0 [47.0; 64.0]	58.0 [38.0; 61.0]	0.72
Male sex, n (%)	22 (62.9)	12 (66.7)	8 (57.1)	0.858
ABO types, n (%)				0.486
A	12 (34.3)	5 (27.8)	5 (35.7)	
B	11 (31.4)	4 (22.2)	5 (35.7)	
O	7 (20.0)	5 (27.8)	0 (0.0)	
AB	5 (14.3)	4 (22.2)	4 (28.6)	
BMI, kg/m^2^, mean ± SD	23.4 ± 4.0	23.9 ± 4.1	23.3 ± 6.3	0.911
Diagnosis, n (%)				0.007
ILD	28 (80.0)	15 (83.3)	8 (57.1)	
BO	2 (5.7)	0 (0.0)	0 (0.0)	
ARDS	5 (14.3)	3 (16.7)	2 (14.3)	
PAH	0 (0.0)	0 (0.0)	4 (28.6)	
Preop rehabilitation, n (%)	26 (74.3)	13 (72.2)	13 (92.9)	0.302
Standing	14 (40.0)	7 (38.9)	2 (14.3)	0.076
Dangling	0 (0.0)	0 (0.0)	1 (7.1)	0.076
Bed exercise	12 (34.3)	6 (33.3)	10 (71.4)	0.076
Transthoracic echocardiography				
LVEF, %, mean ± SD	60.6 ± 7.4	58.3 ± 9.3	63.5 ± 6.0	0.199
E/E′, mean ± SD	7.8 ± 2.5	9.3 ± 4.2	10.9 ± 4.6	0.031
TR Vmax, m/s, mean ± SD	3.3 ± 0.7	3.5 ± 0.6	3.4 ± 0.7	0.785
PGsys (RV-RA), mmHg, mean ± SD	48.0 ± 17.0	49.1 ± 16.2	51.4 ± 17.8	0.842
Lactic acid at ECMO insertion, mmol/L, mean ± SD	2.5 ± 2.2	2.0 ± 0.8	8.2 ± 3.5	<0.000
Lactic acid at ECMO configuration change, mmol/L, mean ± SD	NA	6.1 ± 3.2	NA	NA
VIS at ECMO insertion, mean ± SD	7.5 ± 11.8	6.6 ± 7.4	32.7 ± 20.4	<0.000
VIS at ECMO configuration change, mmol/L, mean ± SD	NA	22.6 ± 19.0	NA	NA
Complications during ECMO, n (%)	13 (37.1)	10 (55.6)	11 (78.6)	0.029
Bleeding	9 (25.7)	8 (44.4)	9 (64.3)	0.037
ECMO pump clot	7 (20.0)	3 (16.7)	4 (28.6)	0.701
ECMO site complication	4 (11.4)	4 (22.2)	3 (21.4)	0.514
CRRT	2 (5.7)	2 (11.1)	5 (35.7)	0.02
Preop ECMO duration, days, median [IQR]	10.0 [3.5; 21.5]	17.0 [9.0; 28.0]	16.0 [13.0; 30.0]	0.09
Day to configuration change, median [IQR]	NA	7.0 [ 4.3; 62.0]	NA	NA
SAPS II at operation day, mean ± SD	35.6 ± 12.0	34.2 ± 12.3	37.9 ± 14.2	0.712
BNP at operation day, mean ± SD	305.9 ± 326.6	538.3 ± 565.9	1,114.5 ± 1,133.0	0.001
Troponin-I at operation day, mean ± SD	0.2 ± 0.3	0.6 ± 1.3	0.7 ± 1.2	0.153

Abbreviations: V-V, Veno-venous; V-A, Veno-arterial; V-AV, Veno-arteriovenous; Oxy-RVAD, right ventricular assist device with an oxygenator; ECMO, extracorporeal membrane oxygenation; RV, right ventricular; BMI, body mass index; ILD, interstitial lung disease; BO, bronchiolitis obliterans; ARDS, acute respiratory distress syndrome; PAH, pulmonary arterial hypertension; LVEF, left ventricular ejection fraction; E/E′, the ratio of early diastolic mitral inflow velocity to early diastolic mitral annular tissue velocity; TR, vmax, maximal tricuspid regurgitation velocity; PGsys(RV-RA), systolic pressure gradient between right ventricle and right atrium; VIS, vasoactive inotropic score; SAPS, the simplified acute physiology score; CRRT, continuous renal replacement therapy; SD, standard deviation; IQR, interquartile range; NA, not applicable.

^a^ Of the 18 patients in the configuration change group, 5 patients were switched to V-A/V-AV ECMO, and 13 patients were switched to Oxy-RVAD.

^b^ Of the 14 patients in the initial V-A ECMO or Oxy-RVAD, group, 5 patients started V-A ECMO, and 9 patients started Oxy-RVAD.

**FIGURE 2:**
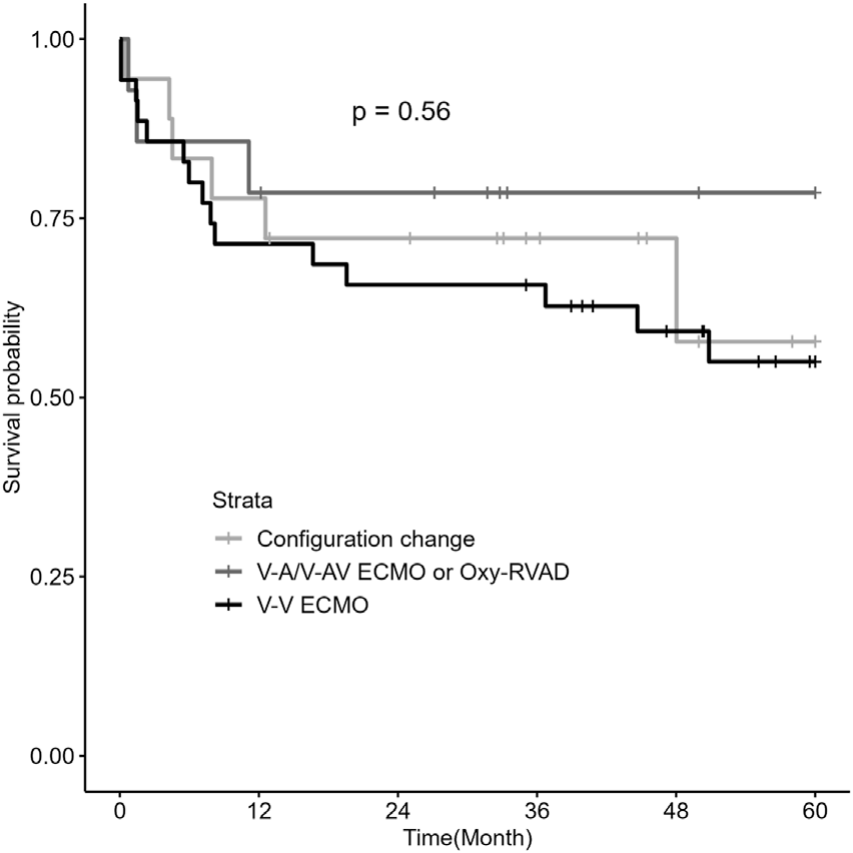
Kaplan–Meier survival analysis by ECMO configurations in patients with RV dysfunction (*n* = 67) Abbreviations: ECMO, extracorporeal membrane oxygenation; RV, right ventricular; V-V, Veno-venous; V-A, Veno-arterial; V-AV, Veno-arteriovenous; Oxy-RVAD, right ventricular assist device with an oxygenator.

Among the three ECMO configuration groups, there were no significant differences in the post-lung transplantation outcomes, including mortality rates, postoperative ECMO application rates, and the grade and percentage of PGD (Table 4). Survival analysis by Kaplan–Meier curve and log-rank test showed that there was no difference in the 5-year survival rate between the V-V ECMO, V-A/V-AV ECMO or Oxy-RVAD, and configuration change groups (Figure 2).

**TABLE 4:** Post-lung transplantation outcomes of the patients stratified according to the ECMO configurations among bridge-to-transplant patients.

	Initial V-V ECMO (*n* = 53)		V-A ECMO or Oxy-RVAD maintained (*n* = 14)	
	V-V ECMO maintained (*n* = 35)	Configuration change to V-A/V-AV ECMO or Oxy-RVAD (*n* = 18)		*p*-value
Postop ECMO application, n (%)	3^a^ (8.6)	1^b^ (5.6)	3^c^ (21.4)	0.302
Postop ICU days, median [IQR]	16.0 [12.0; 31.0]	27.0 [16.0; 48.0]	20.5 [11.0; 43.0]	0.335
Postop hospital days, median [IQR]	69.0 [45.0; 129.5]	85.5 [60.0; 199.0]	92.0 [37.0; 178.0]	0.483
Discharge from hospital n (%)	27 (77.1)	14 (77.8)	11 (78.6)	0.994
30-day vasoactive drug-free day, median [IQR]	27.0 [22.5; 28.0]	27.5 [21.0; 29.0]	27.0 [22.0; 28.0]	0.537
30-day ventilator-free day, median [IQR]	19.0 [6.0; 24.5]	12.0 [0.0; 22.0]	15.5 [0.0; 23.0]	0.534
30-day mortality, n (%)	2 (5.7)	1 (5.6)	1 (7.1)	0.978
90-day mortality, n (%)	5 (14.3)	1 (5.6)	2 (14.3)	0.621
1-year mortality, n (%)	10/35 (28.6)	4/18 (22.2)	3/14 (21.4)	0.819
3-year mortality, n (%)	12/34 (35.3)	5/12 (41.7)	3/8 (37.5)	0.925
5-year mortality, n (%)	15/25 (60.0)	6/8 (75.0)	3/7 (42.9)	0.448
PGD at T72, n (%)				0.886
Grade 0	21 (60.0)	10 (55.6)	8 (57.1)	
Grade 1	4 (11.4)	2 (11.1)	3 (21.4)	
Grade 2	69 (17.1)	5 (27.8)	2 (14.3)	
Grade 3	4 (11.4)	1 (5.6)	1 (7.1)	

Abbreviations: V-V, Veno-venous; V-A, Veno-arterial; V-AV, Veno-arteriovenous; Oxy-RVAD, right ventricular assist device with an oxygenator; ECMO, extracorporeal membrane oxygenation; IQR, interquartile range; PGD, at T72, primary graft dysfunction at 72 h time point.

^a^ Postoperative ECMO, duration in three patients was 3, 3, and 6 days.

^b^ Postoperative ECMO, duration in one patient was 1 day.

^c^ Postoperative ECMO duration in three patients was 6, 11, and 43 days.

**FIGURE 3:**
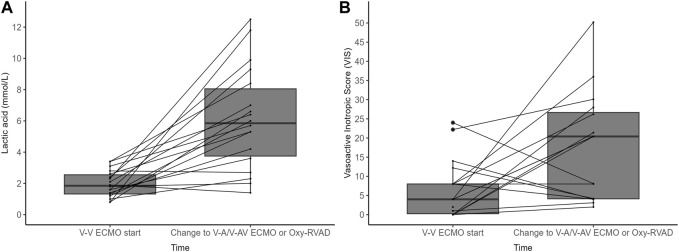
**(A)** Lactic acid level and **(B)** vasoactive inotropic score (VIS) in the configuration change group (*n* = 18) Abbreviations: ECMO, extracorporeal membrane oxygenation; V-V, Veno-venous; V-A, Veno-arterial; V-AV, Veno-arteriovenous; Oxy-RVAD, right ventricular assist device with and oxygenator.

In the configuration change group of V-V ECMO to V-A/V-AV ECMO or Oxy-RVAD groups, the lactic acid level and VIS were significantly different between the time of initial ECMO start and the time of ECMO configuration change (Figure 3; Table 3). The lactic acid level was 2.0 ± 0.8 mmol/L when starting V-V ECMO but it increased to 6.1 ± 3.2 mmol/L when the configuration was changed to V-A/V-AV ECMO or Oxy-RVAD (*p* < 0.001). The baseline VIS was also 6.6 ± 7.4 when V-V ECMO was started but it increased to 22.6 ± 19.0 when the configuration changed (*p* = 0.009).

## Discussion

In this study, we observed that among BTT patients who successfully underwent lung transplantation, 67% exhibited documented RV dysfunction. We compared the three groups of V-V ECMO, configuration changed from V-V ECMO to V-A ECMO or Oxy-RVAD, and V-A ECMO/Oxy-RVAD in RV dysfunction patients. There was no significant difference in the survival rates and other clinical outcomes among the three ECMO configuration groups. Our results suggest that proper ECMO configurations and management are important for survival in patients with RV dysfunction.

In patients awaiting lung transplantation, management of RV dysfunction is very important because preoperative mortality in this group of patients has been reported to be very high if not adequately managed [18, 22]. Moreover, since RV dysfunction improves in most cases after lung transplantation [23, 24], achieving hemodynamic stability by mechanical circulatory support in the perioperative period is a very important issue. A previous study from a high-volume transplant center suggested initiating V-A ECMO rather than V-V ECMO in patients with secondary pulmonary hypertension [1]. In their study, initial V-A ECMO showed higher survival rates for transplantation than initial V-V ECMO [22]. Therefore, the current expert consensus document recommends V-A ECMO as a BTT in selected patients with end-stage pulmonary hypertension who develop right ventricular failure. However, unlike the existing study, in our cohort, V-V ECMO was initially applied in 79% (*n* = 53) of patients with RV dysfunction, and 52% (*n* = 35) of patients eventually underwent lung transplantation via V-V ECMO. Our results also showed no difference in the short- and long-term post-transplant outcomes in the V-V ECMO group compared with the V-A ECMO or Oxy-RVAD groups, although the complication rate was the lowest in the V-V ECMO group. Therefore, we believe that further larger studies are needed in this area.

In current recommendations for rehabilitation while awaiting with BTT, ambulation and rehabilitation should be aggressively pursued to improve bridge success rates and post-transplant outcomes [8]. In this study, the rate of preoperative rehabilitation, especially standing in V-V ECMO patients was not significantly different from that in other configuration groups. This is because when patients with V-V ECMO could not tolerate standing and walking, we changed V-V ECMO to Oxy-RVAD or V-A ECMO to rehabilitate well [17, 18]. Conversely, in cases of hemodynamically stable and compensated RV dysfunction even during mobilization and rehabilitation, proceeding with lung transplantation via V-V ECMO does not affect the post-transplant outcomes. Successful lung transplantation is achieved using only low doses of vasoactive inotropic agents in this group of patients. Finally, our cohort showed similar short- and long-term survival outcomes regardless of ECMO configuration. We suggest that this is because early recognition and prompt adjustment of ECMO configuration in patients who experienced hemodynamic deterioration during V-V ECMO or were unable to tolerate early mobilization and rehabilitation allowed patients with RV dysfunction to survive and successfully undergo lung transplantation.

A noteworthy aspect of this study is the examination of the characteristics in the configuration change group that transitioned from initial V-V ECMO to V-A/V-AV ECMO or Oxy-RVAD. The median duration from V-V ECMO start to configuration change was 7 days. At the time of V-V ECMO insertion, the lactic acid level was 2 mmol/L, and the VIS was 6.6 (equivalent to norepinephrine 0.06 mcg/kg/min), which escalated to a lactic acid level of 6.1 mmol/L and a VIS of 22.6 (equivalent to norepinephrine 0.22 mcg/kg/min) before the configuration change. It is crucial for all BTT patients awaiting lung transplantation to undergo continuous hemodynamic monitoring via arterial line, periodic arterial blood gas analysis, and lactic acid tests to promptly detect any worsening of RV failure and progression to cardiogenic shock. Additionally, transitioning to Oxy-RVAD or V-A ECMO requires involvement from thoracic surgery and ECMO teams; hence, ongoing, close discussions among multidisciplinary teams, including intensivist, ECMO team, and thoracic surgery department, are essential. Failing to change the ECMO configuration in deteriorating hemodynamic situations could lead to progressive organ failure, inhibiting patients from undergoing lung transplantation and potentially leading to mortality.

High doses of vasoactive inotropes were required, and hyperlactatemia may reflect the onset of cardiogenic shock due to RV failure. Patients initially treated with V-A/V-AV ECMO or Oxy-RVAD had significantly higher pre-ECMO lactic acid levels of 8.2 ± 3.5 mmol/L and VIS of 32.7 ± 20.4 (equivalent to norepinephrine 0.33 mcg/kg/min) compared to the other groups. They also experienced the highest rates of bleeding and pump clots, and CRRT application during ECMO was most frequently observed. This suggests a likelihood of accompanying issues, such as DIC or acute kidney injury due to cardiogenic shock. The choice between Oxy-RVAD and V-A ECMO for patients with hemodynamic decompensated RV dysfunction is currently dependent on hospital or physician preference, although the application of Oxy-RVAD is increasing [16]. Oxy-RVAD, requiring left anterior thoracotomy for the placement of the reinfusion cannula in the pulmonary artery, is technically more complex than peripheral V-A ECMO, but it offers several advantages over V-A ECMO, such as enabling systemic circulation with oxygenated blood and showing fewer thromboembolic complications [6, 9]. It also facilitates rehabilitation similar to central V-A ECMO [6, 18, 25]. Hence, its use in patients awaiting lung transplantation is increasingly prevalent. A new device of percutaneous RVAD (Protek Duo, Tandemlife Pittsburgh, PA, United States) could also be a good option for BTT [26]. Further research is necessary regarding the choice between V-A ECMO and Oxy-RVAD in patients with hemodynamic decompensated RV dysfunction.

### Limitations

There are several limitations to this study. First, not all RV parameters were measured. Because there were many parameters to define RV systolic dysfunction, not all parameters (TAPSE, tricuspid annulus DTI S′ velocity, FAC, and RIMP) were measured in each of the patients. However, the RV dysfunction used in our study was verified by a cardiologist after the examination by a cardiac sonographer, lending credibility to the results. Second, this was a single-center study in a setting with a severe lung donor shortage. Many patients are transplanted in status 0 [13, 14]. As a result, the patients are already intubated and on mechanical ventilation due to respiratory failure, and their performance and rehabilitation status may be poorer than those of outpatients, potentially leading to higher long-term mortality rates post-lung transplantation.

In conclusion, in lung transplant candidates with RV dysfunction, the initial ECMO configuration should be determined based on the patients’ current hemodynamic status, including lactic acid levels and the need for vasoactive inotropic support. Patients who maintain stable vital signs with low doses of vasoactive inotropes and low lactate levels even during rehabilitation can continue V-V ECMO until lung transplantation. However, even in patients who are initially stable, an increase in lactic acid levels and the need for vasoactive inotropes may need prompt consideration of a transition to V-A ECMO or Oxy-RVAD. Appropriate changes in ECMO configuration do not lead to an increase in mortality, highlighting the value of a clinically adaptive approach to ECMO management that is customized to each patient’s clinical situation.

## Data Availability

The raw data supporting the conclusion of this article will be made available by the authors, without undue reservation.
